# Metaverse and its impact on medical education and health care system: A narrative review

**DOI:** 10.1002/hsr2.70100

**Published:** 2024-09-24

**Authors:** Faezeh Ghaempanah, Bahar Moasses Ghafari, Darya Hesami, Reza Hossein Zadeh, Rashin Noroozpoor, AmirAli Moodi Ghalibaf, Parsa Hasanabadi

**Affiliations:** ^1^ Student Committee of Medical Education Development, Education Development Center Kurdistan University of Medical Sciences Sanandaj Iran; ^2^ Medicine Faculty Kurdistan University of Medical Sciences Sanandaj Iran; ^3^ Student Research Committee Kurdistan University of Medical Sciences Sanandaj Iran; ^4^ Nuclear Medicine Department Kurdistan University of Medical Sciences Sanandaj Iran; ^5^ Student Committee of Medical Education Development, Education Development Center Birjand University of Medical Sciences Birjand Iran; ^6^ Student Research Committee Birjand University of Medical Sciences Birjand Iran

**Keywords:** health care system, medical education, metaverse, technology

## Abstract

**Background and Aims:**

The metaverse has enormous potential in health care, continuously developing and offering innovative solutions by combining artificial intelligence (AI), augmented reality (AR)/virtual reality (VR), Internet of Medical Devices, and quantum computing technologies. In addition to using virtual platforms to help and boost medical education, familiarity with this platform is necessary to strengthen medical skills and communication with patients in medical sciences in the future.

**Methods:**

We conducted a comprehensive search using keywords and their MeSH synonyms, including “metaverse,” “medical education,” and “health care,” across PubMed, Scopus, and Web of Science. After screening the results, relevant articles were selected to inform the writing of this manuscript.

**Results:**

The metaverse is shaping the future of medical sciences, offering new opportunities for health education, advocacy training, and patient outcome improvement. The combination of real and virtual worlds may advance international relations, facilitate data sharing, increase medical care speed, and reduce infectious diseases. The metaverse, despite its benefits, has some limitations. Only 37% of 15−24‐year‐olds have internet access, and AR/VR glasses are expensive and may cause eye discomfort. It is also a potential risk for medical students, who may need help understanding the limitations of simulations and develop unrealistic expectations. Considering the metaverse as a supplement to clinical practice, not a replacement for supervised training, is crucial. Ethical concerns, data security, privacy, and lack of instructions for education are also issues. However, providing information about the metaverse can increase health care workers' attribution to use it for patient examinations, students' education, and tests.

**Conclusion:**

This paper explores the impact of the metaverse on medical science education and underscores the need to integrate the metaverse into all areas of medical sciences as a supplement to existing evidence.

## 1 INTRODUCTION

The “metaverse” was first used in 1992 by Neal Stephenson. In this literary work, Stephenson mixed real and virtual characters with a three‐dimensional virtual setting known as the metaverse.^1^ In this prosperous cyberspace, people can engage in various activities, from shopping to education. Metaverse is a compound word consisting of two parts: the first part, “meta,” means next or beyond, and the “universe” means the world. In other words, the original word means the world beyond reality. The metaverse provides an environment that combines physical reality and digital virtuality.^2^ Research conducted by Kye et al. categorizes the metaverse into four distinct types: augmented reality (AR), lifelogging, mirror world, and virtual reality (VR). AR connects the physical world with the virtual world, while lifelogging involves the internal reinforcement of individuals' virtual experiences. The mirror world reflects the real world within the virtual realm, and the fourth category encompasses the virtual world itself.^3^ Individuals can participate in various activities within the metaverse, including shopping, playing, socializing, and partying, within a graphically rich cyberspace that closely resembles the real world.^4^

Since the COVID‐19 outbreak, virtual platforms have taken over the central space of various activities, including work, educational activities, remote work, online meetings, distance education, online shopping, and other daily activities that have become an inseparable part of people's lives.^3^, ^5^, ^6^ With its integration of AI, AR/VR, Internet of Medical Devices, quantum computing technologies, and robotics, the metaverse holds immense potential in revolutionizing medical sciences by enhancing surgical precision, enabling therapeutic applications, and more.^4^

The metaverse has benefits in medical education. To establish a qualified medical education, it is essential to entirely examine and engage in conversations about extended reality technologies.^7^ In addition to using virtual platforms to help and strengthen medical education, familiarity with this platform is necessary to strengthen medical skills and communication with patients in the medical sciences in the future. Therefore, familiarity with the types of virtual space platforms that facilitate and accelerate the education process, such as cloud computing, digital twins, VR, AR, 5G, mixed reality (MR), artificial intelligence (AI), and the metaverse, is essential.^3^

While still in its early stages, the metaverse has begun to emerge in medical sciences and health care, emphasizing the need for further research to elucidate its impact on medical education.^8^, ^9^ Consequently, there is a need for more studies exploring how the metaverse can enhance collaborative learning, simulation‐based training, and remote access to medical resources, shaping the future of medical education. In this study, we investigated the usage and effect of the metaverse in the health care system and medical education, including its applications, chances, and limitations. This paper reviews literature that evaluates the effect of metaverse in medical sciences education and adds the need to add metaverse to existing evidence through all the fields of medical sciences.

## 2 METHODOLOGY

Based on the discussion in the introduction section, we identified a significant need to explore and familiarize ourselves with all aspects of the metaverse in medical education and the health care system. To ensure methodological rigor, we followed a structured approach that included three key steps: (1) identifying relevant data to inform the methodological framework, (2) developing a comprehensive methodological framework, and (3) validating, testing, and refining the framework.^10^

Our approach included a detailed assessment of the quality and methodologies of the studies reviewed, evaluating the research designs, sample sizes, and analytical techniques used. This assessment helped us to provide a clearer picture of the strength of the evidence supporting the use of the metaverse in these fields.

In the initial step, we developed a detailed outline for the manuscript. To ensure a comprehensive review, we conducted a search in PubMed, Scopus, and Web of Science databases. Our search strategy was designed to be inclusive, using broad keywords such as “metaverse,” “medical education,” and “health care” along with their MeSH synonyms. Given the novelty of the metaverse, we did not limit the search by publication year, and included all results that were relevant. The search results were then screened through a rigorous process involving title, abstract, and full‐text reviews. The studies were further assessed for their methodological rigor, including an evaluation of potential biases and limitations in their designs.

This study aims to address the following research questions: (1) What are the opportunities presented by the metaverse in medical education and the health care system? (2) What are the challenges and limitations associated with its use?

## 3 WHAT IS THE METAVERSE?

As mentioned, the metaverse began with Nile Stephenson's novel, “Snow Crash.” Initially, it seemed distant from reality, but with the COVID‐19 pandemic and the subsequent surge in virtual interactions for work, science, and education, it has become more tangible.^11^ Nevertheless, actions in the physical world affect our experience in the virtual world and conversely, emphasizing the close link between virtual avatars and the physical environment.^12^

Metaverse is not a single concept; rather, it comprises various technologies, including AR, VR, Internet of things (IoT), 5 G, blockchain, cloud computing, digital twins, MR, and AI.^13^ The Acceleration Studies Foundation introduced this concept in 2006, identifying four dimensions: AR, lifelogging, mirror world, and VR. These dimensions are divided into two axes: augmentation versus simulation and intimate versus external.^3^

The first technology, *augmented reality (AR)*, overlays virtual elements onto the real world, resembling viewing reality through smart glasses.^14^, ^15^ The second technology, lifelogging, pertains to documenting, retaining, and disseminating daily occurrences and details concerning individuals and objects. Notably, it mentions maintaining medical records in any format, which can be shared between doctors specializing in different fields, and provides comprehensive information for the patients' medical records.^3^, ^16^

*Mirror world* replicates real‐life scenarios in a virtual environment to facilitate experiential learning, gaining attention particularly post‐COVID‐19.^3^ The last technology, VR immerses users in a 3D virtual world, often utilized in medical education with realistic graphics and fast communication tools.^11^, ^16^, ^17^ Platforms like Zepeto and Roblox offer interactive social and creative experiences.^18^ Head‐mounted displays (HMDs) provide a stereo scene by presenting separate images to each eye, dynamically adjusting based on head position and gaze direction.^19^

The link between the metaverse and the internet has evolved over distinct phases: the portal era, the search or social era, and the digital smart Internet era, which integrates physical and virtual worlds seamlessly, facilitating global access to educational resources.^11^ The link between the metaverse and the internet typically benefits education and grants students’ worldwide access to the finest educational materials from any location. This facilitates providing high‐quality, standardized education to all learners, irrespective of their location or the time at which they are learning.^20^ In addition to medical education, the metaverse also finds applications in assisting patients' treatment. Leveraging the internet and intelligent metaverse technologies, individuals living in remote areas can access top‐tier medical professionals. Patients can readily share test results with doctors using the internet, headsets, and other smart devices. Evidently, the metaverse's primary objectives are to supplement traditional medical sciences offerings rather than replace them.^21^, ^22^, ^23^

AI is regarded as a field of science and engineering focused on developing intelligence and behavioral capabilities in machines or computers.^24^ AI is a subcategory of the metaverse. Despite the expansions that AI has received in the field of medical functions, however, one explicit limitation of AI's bedside performance is the empathy, high‐level conversation, and body language that are essential for replacing human interactions.^7^

## 4 APPLICATION OF THE METAVERSE IN THE HEALTH CARE SYSTEM

**Figure 1:**
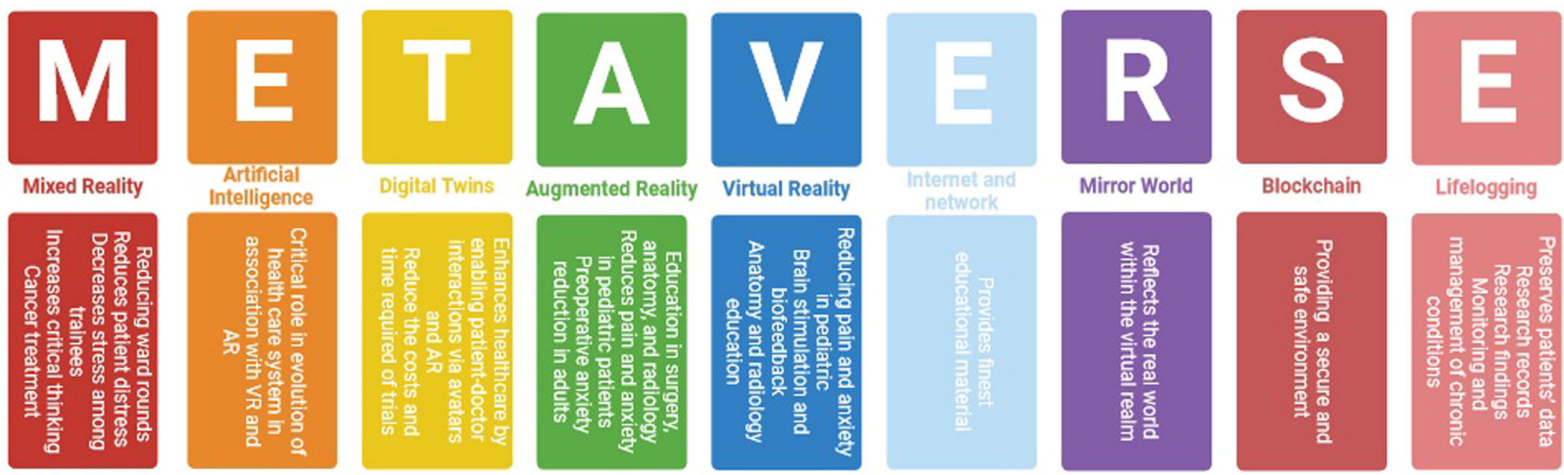
Usage of metaverse subsidiaries in medical education and health care system.

Emerging technologies are rapidly evolving the medical sciences,^25^ with digital services identified as groundbreaking element within the health care.^4^ Following the COVID‐19 pandemic, face‐to‐face interactions were restricted, prompting the extensive uptake of remote care and communication.^26^ In a study conducted by Marr et al. which revealed that three new technologies, such as telepresence, digital twinning, and blockchain, possess considerable potential to significantly impact health care, treatment provision, cost reduction, and the overall improvement of patient outcomes.^27^ The metaverse is constructing many opportunities in the health care sector (Figure 1). In this section, we demonstrate some usages of metaverse.

The use of virtual worlds, such as *second life*, has been explored in the context of medical and health education. *Second life* is a simulated environment used to build environments where people can gain information and interact. It has been found to have many technically advantageous characteristics.^28^, ^29^ Virtual worlds like *Second life* have significantly impacted health care, treatment provision, and cost reduction. The COVID‐19 pandemic has also led to the widespread adoption of remote care and contact, further emphasizing the potential of digital services in health care.^30^ Thus, the combination of virtual worlds and emerging technologies is shaping the future of medical sciences, offering new opportunities for health education, advocacy training, and the improving patients' outcomes.

Within the metaverse, digital twins enhance health care by enabling patient‐doctor interactions via avatars and AR,^31^ transforming care, mental health support, and medical training.^32^ Digital twins in health care can create an immersive and personalized health care experiences for users, enabling them to interact, socialize, and navigate virtual health care settings and clinical encounters.^33^

Lifelogging technologies, such as wearable devices and sensors, provide real‐time patient health data, enabling health care providers to monitor and manage chronic conditions more effectively. The potential of metaverse and lifelogging technologies for health care is vast. However, there are still challenges to their adoption, such as the need for more comprehension and resistance to innovative solutions. However, the metaverse and lifelogging technologies are set to revolutionize health care, offering unprecedented opportunities to enhance patient care, medical training, and research.^34^

MR has some usage in health professions. MR headsets are used in surgery. The basis of MR headsets is context guidelines and ward rounds. Using these headsets, the surgeon can view and check the bedded patient's vital signs through the visual display. One of the outcomes of using this innovation is to decrease the time of ward rounds by a third (43 min) and reduce the number of individuals in the ward. The overall experience with MR is positive, and the main benefits seem to be related to enhanced efficiency. However, there are limitations primarily related to conditions associated with head‐mounted displays.^35^, ^36^ Another MR technology is HoloLens. This technology allows for real‐time visualization and guidance during the procedure, improving the accuracy and efficiency of cancer treatment. Radiologists can also use holographic images from Microsoft HoloLens to determine the tumor's exact location so that the biopsy needle hits the tumor more accurately and also finds a suitable direction for X‐rays to hit the tumor.^37^

The combination of the real and virtual world may advance international relations of clinicians, facilitate data sharing, increase the speed of receiving medical care, video conferencing medical consultations, reduce the risk of infectious diseases for treatment staff and patients, and supervise the delivery of medicine to the patient and the course of recovery with new technologies.^37^ An example of this technology is OptiVu. OptiVu is a holographic software integrating the real and digital worlds, which, with the support of Microsoft HoloLens, allows the patient to receive consultations, advice, personalized care, and diagnosis and treatment of their disease.^38^

VR technology is effective in reducing pain and anxiety in pediatric patients who undergo medical procedures such as vaccinations, intravenous injections, scratch repair, and changing dress for burn wounds.^39^, ^40^ KindVR a research‐based company, offers customized VR therapies to help pediatric patients in managing pain and stress related to medical conditions.^41^ Collaborating with hospitals in the US and Canada, KindVR conducts over 10 trials across various fields, including sickle cell disease, cancer treatments, and preoperative stress management. Moreover, VR applications in brain stimulation and biofeedback enhance treatment precision and personalized therapy based on patient responses.^42^ Recent studies have shown that VR technology reduces preoperative and operative anxiety and stress and enhances preparedness in adults undergoing elective surgeries and cesarean sections. Dream sites, utilized in VR simulations, serve as distractions during childbirth, fostering relaxation and comfort.^43^, ^44^

Machine learning and sophisticated AI‐driven advancements like augmented/VR, the metaverse, and language models are becoming critical focal points in health care's digital evolution. Such inventions aim to enhance education, diagnosis, and therapeutics in cytopathology, transforming it into a highly digitalized realm.^45^

Metaverse has transformed the pharmaceutical industry. The pharmaceutical industry is engaged in exploring, developing, producing, and recovering medicines and their administration to patients to treat, vaccinate, or relieve their symptoms. Trials are the most expensive and time‐consuming phases of medicine research. They are developing a treatment account for 60% of the medical expenses. The extraordinary potential of digital twins to drastically reduce the costs and time required for tests is known. High‐motivated pharmaceutical companies are looking for new technologies.^46^ One of the efforts made in this field can be called Vis‐Mol. It supports Microsoft's AR‐based HoloLens technology. It allows pharmacists to see the molecule structure of the drug in the real environment.^47^

In psychology, Rothbaum et al. conducted the first VR study in 1995. The subject of their study was acrophobia among students, which led to the emergence of a new window of research to influence VR in the face of treatment of obsessive‐compulsive disorder and similar disorders.^48^, ^49^ Extended reality (XR) headsets improve mental health and treat phobia by directly facing the patient in controlled conditions with intimidating stimuli.^50^ Over time, individuals who experience chronic diseases also tend to experience mental health challenges. Metaverse instills a sense of support and importance in them by creating virtual support bases and formations and promotes their mental and physical health.^51^

## 5 METAVERSE IN MEDICAL EDUCATION

During the COVID‐19 pandemic, traditional classrooms were disrupted,^52^, ^53^ leading to a decline in the quality of education and learning. Factors contributing to this decline included decreased students' motivation and the challenges associated with virtual learning, with medical education being not exception. However, the pandemic also presented opportunities for innovation and growth in medical education. The emergence of the metaverse significantly enhanced reality and VR, offering a high‐potential environment capable of driving the education system towards development and progress, addressing significant limitations in student learning.^54^ (Figure 1).

Visualizing actual images in these virtual spaces helps students better learn and investigate the health science content that the metaverse creates in these spaces.^55^ It offers vast potential in health care education, offering interactive and inclusive programs tailored to students’ preferences.^56^ Immediate and personalized feedback in the Metaverse allows learners to identify weaknesses and improve accordingly.^42^, ^57^ This feedback enables trainees to identify their weaknesses and then improve and adjust their approach accordingly.

Logsit and Nomie are two popular tools for lifelogging. Lifelogging can provide a more personalized and empowering learning experience, provide valuable data that can be used to improve the quality of medical education and support the goal of making lifelong learning a reality for physicians.^58^, ^59^ The use of this technology in education is to preserve patients' data, research records, and research findings.^3^, ^16^, ^59^

AR provides a large amount of knowledge, both textual and audio‐visual, in the field of medical education.^60^ For example, the use of smart glasses with AR technology in medical education can reduce the gap and difficulty caused by learning anatomy to a great extent, so putting these glasses on the eyes will help surgeons, radiologists, and specialists in other branches of medical science to apply anatomy to the human body quickly.^4^ Additionally, in the United Kingdom, there is a t‐shirt created with AR technology, enabling users to view the internal structure of the human body, akin to what is typically studied in anatomy.^16^

Metaverse gives its users a high degree of freedom that expands students' autonomy in learning.^61^ AR and VR are good tools for teaching students, especially in anatomy and radiology. At the same time, the metaverse has shown more potential for clinical education, and its most significant impact is in revolutionizing simulation‐based learning in surgical education.^62^ Learners can gain practical surgical experience in a risk‐free environment by eliminating patient risk; almost inclusive surgical training can be accessible even to first‐year medical students.^63^ The ongoing development of metaverse‐compatible technologies has shown the potential to revolutionize clinical skills training strategy. Metaverse can be used for remote surgical assistance to revolutionize clinical skills.^64^ In the surgery, a study showed that VR simulation effectively taught students to perform total knee arthroplasty (TKA). Students trained using VR simulation performed significantly better in a TKA‐simulated job compared to those who did not use simulation.^65^ Besides, AR offers enormous potential in health care education and practice. Metaverse allows surgeons to remote mentor and locate hard‐to‐find breast cancers using AR.^66^ In addition, there are efforts to make some pulse pressure waveforms for medical palpation training that provide consistent and accurate simulations for educators.^67^

AR, VR, and MR have previously shown their capabilities in medical environments, including improved surgical precision, reduced patient distress during medical procedures, decreased stress among trainees, and increased critical thinking.^68^, ^69^

### 5.1 Applications of the metaverse in privacy

While using the metaverse offers numerous patient advantages, it also presents particular challenges. It is crucial to consider privacy and security concerns when discussing the use of data generated by the metaverse.^70^ Metaverse providers' policies must address the potential harms that social media and earlier mental health‐based technologies have caused. Information collected by the metaverse could be used for shaping consumer beliefs and behaviors highlighting the for clear legal frameworks, especially for medical purposes.^71^, ^72^ A recent study by Benrimoh et al. emphasized the importance of obtaining approval from medical regulatory systems like HIPAA and PIPEDA before using metaverse technologies for medical purposes. HIPAA and PIPEDA are medial protocols established to safeguard the privacy and integrity of health information in the United States and Canada, respectively.^70^

It is generally understood that databases collecting and storing data from health care systems should maintain confidentiality to avoid unauthorized disclosure of patient information. Moreover, inadequate data management can compromise physical and mental health activities and contribute to a widespread lack of trust in society. Consequently, treatment and care systems are responsible, especially in psychiatry, where VR/AR technologies are employed, must prioritize patient privacy protection to foster mutual trust.^72^, ^73^

Cyberattacks and data breaches can compromise the privacy and security of patient data. Information breaches occur when personal or organizational information or data is stolen, read, or shared by an unauthorized person through a device. XR systems may show weakness against cyberattacks and hacking, necessitating decisive security measure to prevent theft and unauthorized access to patient information.^74^

Federal Learning (FL), an emerging branch of AI, is developing as a published template, often utilizing data collected through the IoT. Recent advances in IoT have led to the evolution of the Internet of Medical Things (IoMT), which finds applications across various fields of medicine. IoMT data security is crucial for modern and intelligent care systems, with FL playing a role in diagnosis, patient monitoring, medical education, infectious diseases, and drug discovery.^75^, ^76^

### 5.2 Challenges and limitations and conclusion

Despite its numerous benefits, the metaverse also presents several limitations and challenges that need to be addressed. One significant obstacle is the limited internet access among youths aged 15–24, with only 37 percent having access to the internet according to a report by United Nations Children's Fund and International Telecommunication Union.^77^

Due to novelty of metaverse, this new technology is already unknown to many people.^78^, ^79^ Many individuals lack understanding about technology, and technophobia further contributes to resistance to using metaverse.^80^ However, accepting traditional society is connected to this technology's familiarity, efficiency, and widespread adoption.^78^ In addition, user satisfaction emerged as a critical determinant for intending to use the metaverse.^81^ Individuals receptive to uncertainty and willing to accept innovation demonstrated a greater propensity toward utilizing the metaverse for medical education.^82^

Furthermore, AR/VR glasses are needed for a truly inclusive experience. However, glasses have a high financial cost and may lead to complications, including discomfort, pressure, fatigue of the eye, and blurred vision. Additionally, the use of metaverse technology in medical settings presents potential risks. One concern is that surgical students and trainees may not fully understand the limitations of simulations and could develop unrealistic expectations about their abilities in real surgical scenarios. Therefore, viewing the metaverse as a supplement to clinical practice rather than a replacing supervised surgical training is essential. It is crucial to thoroughly assess this emerging technology's advantages, disadvantages, and potential risks. To ensure that health care professionals are prepared to deliver excellent care, metaverse technology must be responsible, supervised, and ethical, alongside formal surgical training.^63^, ^83^ Besides, medical science and technology advancements have raised ethical concerns in new medical scenarios.^84^ Data security and privacy, the imbalance between the real and virtual world, and the absence of instructions for education in the metaverse are challenges that need attention.^85^

While the metaverse can be a platform for communication development, it should be noted that it only displays the part of users who want to share it, and not all aspects of a person are seen. In addition, the low rate of privacy violations in the virtual world is an undeniable problem.^85^ The freedom of the metaverse and the broader world with fewer rules than the real world has brought us can provide a platform for dangerous crimes, so its use for young people and adolescents should be done with full awareness of this field.^3^ One of the most critical problems due to Torres navigation that the use of the new Torres navigation in the clinic has brought us is the inability to communicate directly with the patient and to attend the patient's clinic, which is necessary for clinical medicine.^86^ Prolonged use of VR headsets can lead to headaches, dizziness, nausea, and vision problems, especially myopia. The expansion of the virtual platform's use endangers users' cybersecurity and increases the incidence of various scams on the platform.^78^

Usually, using new technologies like the metaverse has been associated with challenges to persuade audiences. Recent studies have shown that providing information about the metaverse increases health care workers' ability to use this new technology for patients' examinations, students' education, and tests.^31^ In addition, students' interest in using metaverse technology was significantly influenced by their enjoyment of use, innovativeness, ease of use, turn learning into fun with the visual presentation of metaverse, and perceived usefulness.^53^, ^69^ Likewise, the students' inclination towards using metaverse technology was significantly impacted by their pleasure in its usage, creativity, user‐friendliness, and perceived utility.

To guarantee the practicality and effectiveness of the metaverse, it is necessary to consider the labor costs involved in its development.^87^ A study conducted by Delshad et al. explored the use of VR therapy for pain management in patients and discovered that while these technologies could relieve some financial obligations in hospitals, they would also incur additional costs.^88^ Besides, having good data security and protection, a user‐friendly environment, increasing youths' access to laptops, and preparing instructions for using this new technology may improve its efficacy. Blockchain technology can provide a secure and safe environment for users. This technology's characteristics, such as immutability, traceability, and transparency, are factors of inaccessible security for data that can be used in the health care system,^89^, ^90^ and by providing a secure environment, patients' confidence in using this technology increases.^91^

In conclusion, the metaverse can significantly affect medical education and the health care system. The metaverse has the potential to revolutionize medical education and the health care system by providing enhanced learning experiences, telemedicine, improving patient care, and overcoming geographical barriers. It can offer immersive learning experiences, improve medical training, enhance patient care, and advance medical education. Also, using this new technology to stimulate conditions for educators may reduce medical errors and increase their critical thinking. However, it is essential to address the challenges and limitations of implementing the metaverse to ensure its successful adoption in medical education and health care settings. With the metaverse's ethical concerns, challenges, and limitations, instructions should be written for using this technology in the health care system and medical education. Based on our findings, future studies should focus on improving user‐friendliness, low‐cost devices, availability, student engagement, and incorporating of metaverse technologies in health care environments while addressing confidentiality, protection, and ethical deliberations.

## AUTHOR CONTRIBUTIONS

Parsa Hasanabadi designed the search strategy and outline. Parsa Hasanabadi and Bahar Moasses Ghafari screened the results and selected the articles for this article. The figure was designed by Parsa Hasanabadi. All authors contributed to writing the manuscript, and Parsa Hasanabadi, Amir Ali Moodi Ghalibaf, and Bahar Moasses Ghafari reviewed it. Bahar Moasses Ghafari, Darya Hesami, and Reza Hossein Zadeh contributed equally to the writing of the manuscript. All authors have read and approved the final version. Parsa Hasanabadi had full access to all the data in this study and takes complete responsibility for the integrity of the data and the accuracy of the data analysis.

## CONFLICT OF INTEREST STATEMENT

The authors declare no conflict of interest.

## ETHICS STATEMENT

This study has the approvement of Research Ethics Committees of Kurdistan University of Medical Sciences. The ethical ID is IR.MUK.REC.1402.255.

## TRANSPARENCY STATEMENT

The lead authors Amir Ali Moodi Ghalibaf, Parsa Hasanabadi affirm that this manuscript is an honest, accurate, and transparent account of the study being reported; that no important aspects of the study have been omitted; and that any discrepancies from the study as planned (and, if relevant, registered) have been explained.

## Data Availability

Data sharing not applicable to this article as no datasets were generated or analyzed during the current study.
